# The last universal common ancestor between ancient Earth chemistry and the onset of genetics

**DOI:** 10.1371/journal.pgen.1007518

**Published:** 2018-08-16

**Authors:** Madeline C. Weiss, Martina Preiner, Joana C. Xavier, Verena Zimorski, William F. Martin

**Affiliations:** 1 Institute of Molecular Evolution, Heinrich Heine University, Düsseldorf, Germany; 2 Instituto de Tecnologia Química e Biológica, Universidade Nova de Lisboa, Oeiras, Portugal; Warwick Medical School, UNITED KINGDOM

## Abstract

All known life forms trace back to a last universal common ancestor (LUCA) that witnessed the onset of Darwinian evolution. One can ask questions about LUCA in various ways, the most common way being to look for traits that are common to all cells, like ribosomes or the genetic code. With the availability of genomes, we can, however, also ask what genes are ancient by virtue of their phylogeny rather than by virtue of being universal. That approach, undertaken recently, leads to a different view of LUCA than we have had in the past, one that fits well with the harsh geochemical setting of early Earth and resembles the biology of prokaryotes that today inhabit the Earth's crust.

## Introduction

The very earliest phases of life on Earth witnessed the origin of life and genetics from the elements. There was a time when there was no life on Earth, and there was a time when there were DNA-inheriting cells. The transitions are hard to imagine. Some dates and constraints on the order of events helps us to better grasp the problem. The Earth is 4.5 billion years (Ga) old [1]. By about 4.4 Ga, the moon-forming impact turned the Earth into a ball of boiling lava [1]. Magma oceans with temperatures over 2,000°K forced all water from early accretion into the gas phase and converted all early accreted carbon to atmospheric carbon dioxide (CO_2_) [1,2]. By 4.2 to 4.3 Ga, the Earth had cooled sufficiently enough that there was liquid water [3]—those first oceans were about twice as deep as today's [1,2]. Only later, hydrothermal convection currents started sequestering water to the primordial crust and mantle, which today bind one extra ocean volume [4,5]. The first signs of life appear as carbon isotope signatures in rocks 3.95 billion years of age [6]. Thus, somewhere on the ocean-covered early Earth and in a narrow window of time of only about 200 million years, the first cells came into existence. Because the genetic code [7] and amino acid chirality [8] are universal, all modern life forms ultimately trace back to that phase of evolution. That was the time during which the last universal common ancestor (LUCA) of all cells lived.

### LUCA, the tree of life, and its roots

LUCA is a theoretical construct—it might or might not have been something we today would call an organism. It helps to bridge the conceptual gap between rocks and water on the early Earth and ideas about the nature of the first cells. Thoughts about LUCA span decades. Various ideas exist in the literature about how LUCA was physically organized and what properties it possessed. These ideas are traditionally linked to our ideas about the overall tree of life and where its root might lie [9–18]. Phylogenetic trees are, however, ephemeral. It is their inescapable fate to undergo change as new data and new methods of phylogenetic inference emerge. Accordingly, the tree of life has been undergoing a great deal of change of late.

**Fig 1:**
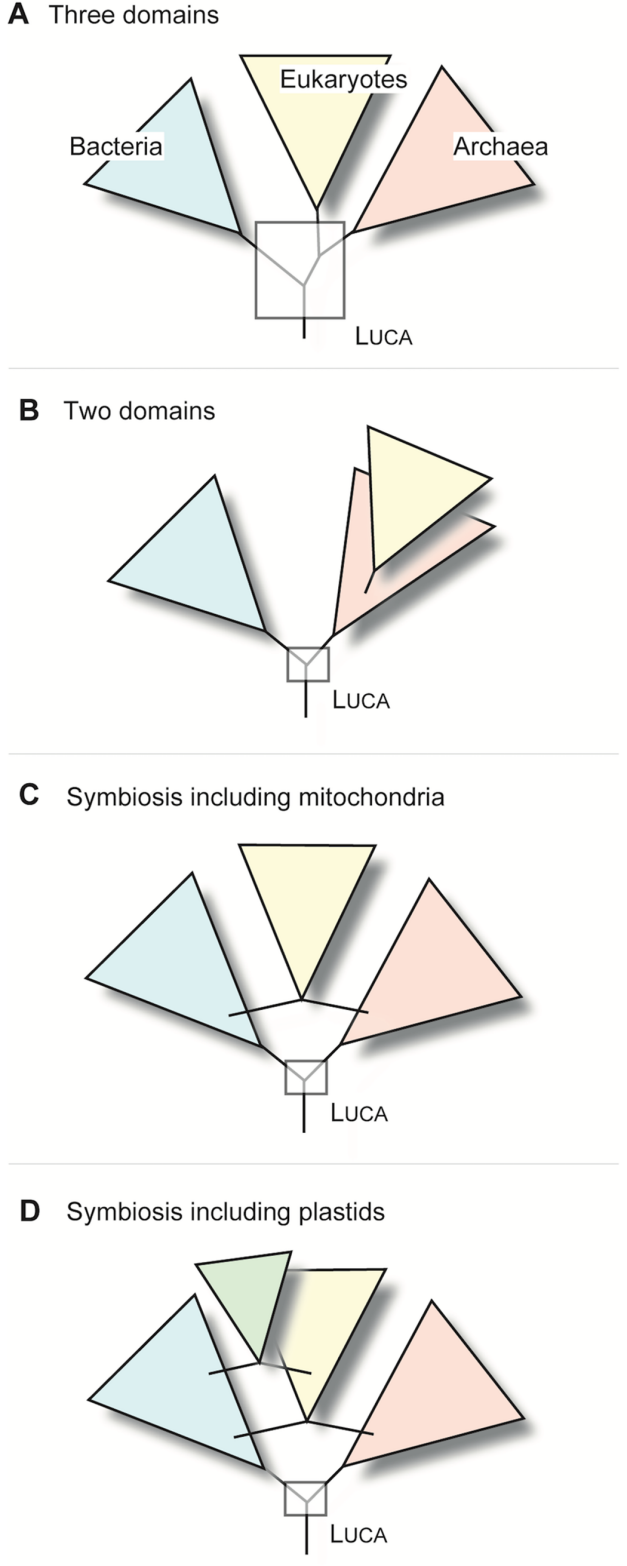
Different views on domain relationships in the tree of life. (A) The three-domain tree: based on rRNA phylogeny, the three domains were of equal rank. (B) The two-domain tree: modern trees show eukaryote cytosolic ribosomes branching within the diversity of archaeal ribosomes. (C) As eukaryotes are not just grownup archaea, the eukaryote ancestor possessed mitochondria. If mitochondrial-derived genes are taken into account, the tree is no longer a bifurcating graph. (D) If plastids are included, the tree becomes even less tree-like because the photosynthetic lineages of eukaryotes also acquired many genes from the plastid ancestor [23].

The familiar three-domain tree of life presented by ribosomal RNA [19] depicted LUCA as the last common ancestor of archaea, bacteria, and eukaryotes (Fig 1A). In that framework, efforts to infer the gene content, hence the properties of LUCA, boiled down to identifying genes that were present in eukaryotes, archaea, and bacteria. When the first genomes came out, there were a great many such investigations [20–22], all of which were confronted with the same two recurrent and fundamental problems: 1) How are the three domains related to one another so that gene presence patterns would really trace genes to LUCA as opposed to another evolutionarily more derived branch? 2) Does presence of a gene in two domains (or three) indicate that it was present in the common ancestor of those domains, or could it have reached its current distribution via late invention in one domain and lateral gene transfer (LGT) from one domain to another?

The first problem (the root of the domains) has been the subject of much recent work. Phylogenetic advances and new metagenomic data are changing the three-domain tree [19] into a two-domain tree [24,25]. This is partially a development around phylogenetic methods [24,26–28] but also entails new archaeal lineages that are now being assembled from metagenomic data and that appear to be more closely related to the host that acquired the mitochondrion than any other archaea known so far [29,30]. The two-domain tree showing an "archaeal origin of eukaryotes" [24,28] (Fig 1B) only tells part of the story, though, because eukaryote genomes harbor more bacterial genes than they do archaeal genes by a factor of about 3:1 [31–33], and those bacterial genes furthermore trace to the eukaryote common ancestor [23]. Eukaryotes are not just big, complex archaea; genomically and at the cellular level, they are true chimeras in that they possess archaeal ribosomes in the cytosol and bacterial ribosomes in mitochondria (Fig 1C) [34]. That polarizes cellular evolution in the right direction (there were once debates about eukaryotes being ancestral [10,13,14,22], as discussed elsewhere [35–37]) and identifies eukaryotes as latecomers in evolution, descendants of prokaryotes [38].

Current versions of the two-domain tree focus on the phylogeny of a handful of about 30 genes, mostly for ribosomal proteins (Box 1) but also on sequences from metagenomic samples. The metagenomic studies [29,30] have generated debate. Metagenomic data can bring forth alignments of genes that were sequenced accurately but have the wrong taxonomic label. For example, Da Cunha and colleagues [39] reported that published trees [29] hinge upon a strong signal stemming from one gene out of 30 and that the gene in question (an elongation factor [EF2]) might not be archaeal but eukaryotic instead. Spang and colleagues [40] defended their tree, eliciting more debate [41]. Errors can also occur in the assembly pipeline [42] en route to alignments [43], independent of contamination. Notwithstanding current debate about metagenomics-based trees of life [24,39,40,42,43], we should recall that rRNA itself produces the two-domain tree when various tree construction parameters are employed [24,26,27]. Both data and methods bear upon efforts to construct trees of life. It remains possible that some aspects of domain relationships might never be resolved to everyone's satisfaction—even the endosymbiotic origin of mitochondria is still debated [37]. But the bacterial origin of mitochondria and their presence in the eukaryote common ancestor [44–47], together with the tendency of eukaryotes to branch within archaeal lineages as archaeal lineage sampling [29,30,48] and phylogenetic methods [24,26,27,32] improve, indicates that eukaryotes arose from prokaryotes and that genes that trace to the common ancestor of archaea and bacteria trace to LUCA.

**Box 1. The tree of 1% and the tree of everything else**

A traditional approach to LUCA has been to simply look for the genes that are present in all genomes. That is easy enough, but the results are sobering. What one finds is a collection of about 30 genes, mostly for ribosomal proteins, telling us that LUCA had a ribosome and had the genetic code, which we already knew [63–65]. That collection of about 30 genes has been in use for about 20 years as concatenated alignments to make trees of lineages based on larger amounts of data than rRNA sequences have to offer [66]. The genes that are present in all lineages (or nearly all) inform us about how LUCA translated mRNA into protein, but they do not tell us about how or where LUCA lived. That information concerns ecophysiology, and physiological traits are not universally conserved—they are what makes microbes different from one another. One can relax the criteria of universal presence a bit and allow for some gene loss in some lineages, in which case, one finds about 100 proteins that are nearly universal [67]. If one puts no size constraints on LUCA's genome and allows loss freely, then all genes present in at least one archaeon and one bacterium trace to LUCA, making it the most versatile organism that ever lived [51]. New insights about microbial phylogeny are emerging from concatenated alignments [24,29,30,42,48,68]. But one has to take care not to get genes from different lineages mixed up, which can be difficult when metagenomes are involved [39,43]. Furthermore, data concatenation has its own pitfalls [66,69,70]. Most modern concatenation studies [29,30,48] employ site-filtering methods in an attempt to remove "noise," but even sites that look "noise free" can still contain bias and conflicting data [63]. Another problem is that popular methods of phylogenetic inference produce inflated confidence intervals on phylogenies and branches [71]. Trees of ca. 30 concatenated proteins are no more immune to phylogenetic error than rRNA is and are prone to additional kinds of error [72]. As it relates to LUCA, regardless of the backbone tree, we still need to know what all proteins say individually about their own phylogenies.

The second problem (how much LGT has there been between domains) that has impaired progress on LUCA has arguably been more difficult to resolve than the rooting issue. If a given gene is present in bacteria and archaea, was it present in LUCA, or could it have been transferred between domains via LGT? As one important example, early studies pondered the presence of bacterial type oxygen (O_2_)-consuming respiratory chains in archaea [21]. Does that mean that archaea are ancestrally O_2_ consumers? As O_2_ is the product of cyanobacterial photosynthesis [49] if we presume archaeal O_2_ respiration to be an ancestral trait of archaea, it means that archaea arose after cyanobacteria, which are only about 2.5 billion years old and gave rise to plastids (Fig 1D) only about 1.5 billion years ago [50]. If ancestral archaea were oxygen respirers, and ancestral bacteria were too, suddenly neither the two-domain tree nor the three-domain tree (Fig 1) make sense because everything is upside down and rooted in cyanobacteria. Similar issues are encountered for many genes and traits [51]. Lateral gene transfer among prokaryotic domains helps to resolve such problems because it decouples physiology (ecological trait evolution) from phylogeny (ribosomal lineage evolution) [52], but it also makes genes more difficult to trace to LUCA.

### Has lateral gene transfer obscured all records?

That takes us to the other extreme. If all genes have been subjected to LGT, as some early claims had it [53], then LUCA would be altogether unknowable from the standpoint of genomes. Early archaeal genomes did indeed uncover abundant transdomain LGT [54], and many bacteria to archaea transfers can be correlated to changes in physiology [55], including the transfer of O_2_-consuming respiratory chains [55–58]. For reconstructing LUCA, the issue boils down to determining i) which genes are present in both archaea and bacteria, ii) which of those are present in both prokaryotic domains because of LGT between archaea and bacteria, and iii) which are present because of vertical inheritance from LUCA. For that, there are currently two methodological approaches. One involves making a backbone reference tree from universally conserved genes that are present in each genome—the tree of 1% [59] (see Box 1)—plotting all gene distributions on the tips of that tree, and then estimating which genes trace to LUCA on the basis of various assumed gain and loss parameters [60–62]. If we permit loss freely, many genes will trace back to LUCA; if we assume many gains, LUCA will have few genes [61]. Constraining ancestral genome sizes helps constrain estimates of which genes trace to LUCA [61] but only if we assume that the tree of each gene is compatible with the reference tree, which is a very severe assumption and unlikely to be true. Each gene has its own individual history (Box 1).

### Each gene records its own evolutionary history

If any protein-coding genes have been vertically inherited from LUCA, their trees should reflect that. To find such trees, one has to make all trees for all proteins, meaning one has to make clusters for all protein-coding genes from large numbers (thousands) of sequenced genomes. Clusters correspond to "natural" protein families of shared amino acid sequence similarity. Given modern computers, making alignments for all such clusters and making maximum likelihood trees for all such alignments is a tractable undertaking. Because LGT among prokaryotes is a real and pervasive process shaping prokaryote genome evolution [55,58,73–77], one has to treat each gene as a marker of its own evolution, not as a proxy for other genes or as a function that is subordinate to ribosomal phylogeny.

Genes that are present in several bacterial lineages and one archaeal lineage (or vice versa) might have been present in LUCA, but they might also have been the result of LGT [55,56,58]. An example illustrates how each gene tree can discriminate between vertical inheritance from LUCA and interdomain LGT. A recent study investigated the 6.1 million proteins encoded in 1,981 prokaryotic genomes (1,847 bacteria and 134 archaea) [78]. The proteins were clustered using the standard Markov Cluster (MCL) method [79]. The first step in that procedure is a matrix containing 18.5 trillion elements ((n^2^-n)/2), each element corresponding to a pairwise amino acid sequence comparison. The clustering of such a matrix requires substantial computational power and is aided by the availability of several terabytes of memory in a single machine. The MCL algorithm samples the distribution of values in the matrix and then starts removing the weak edges, with the value of "weak" being specified by the user. Two kinds of thresholds are typically used in MCL clustering: BLAST e-values and amino acid identity in pairwise alignments.

When the goal of clustering is to make alignments and trees, our group has found that a clustering threshold of 25% amino acid identity is a good rule of thumb. At lower thresholds, amino acid identity starts to approach random values and generates random errors in alignments [80], carrying over as erroneous topologies in trees [81]. That is why Russell F. Doolittle coined the term "twilight zone" for amino acid identity at or below the 20% range [82,83]. Of course, many proteins or domains that clearly share a common ancestry by the measure of related crystal structures do not share more than a random amino acid sequence identity [84]. Such ancient folds will fall into separate clusters at the 25% identity threshold and might thus generate false negatives when it comes to presence in LUCA (but see next section).

### From thousands of clusters and trees, a handful remain

**Fig 2:**
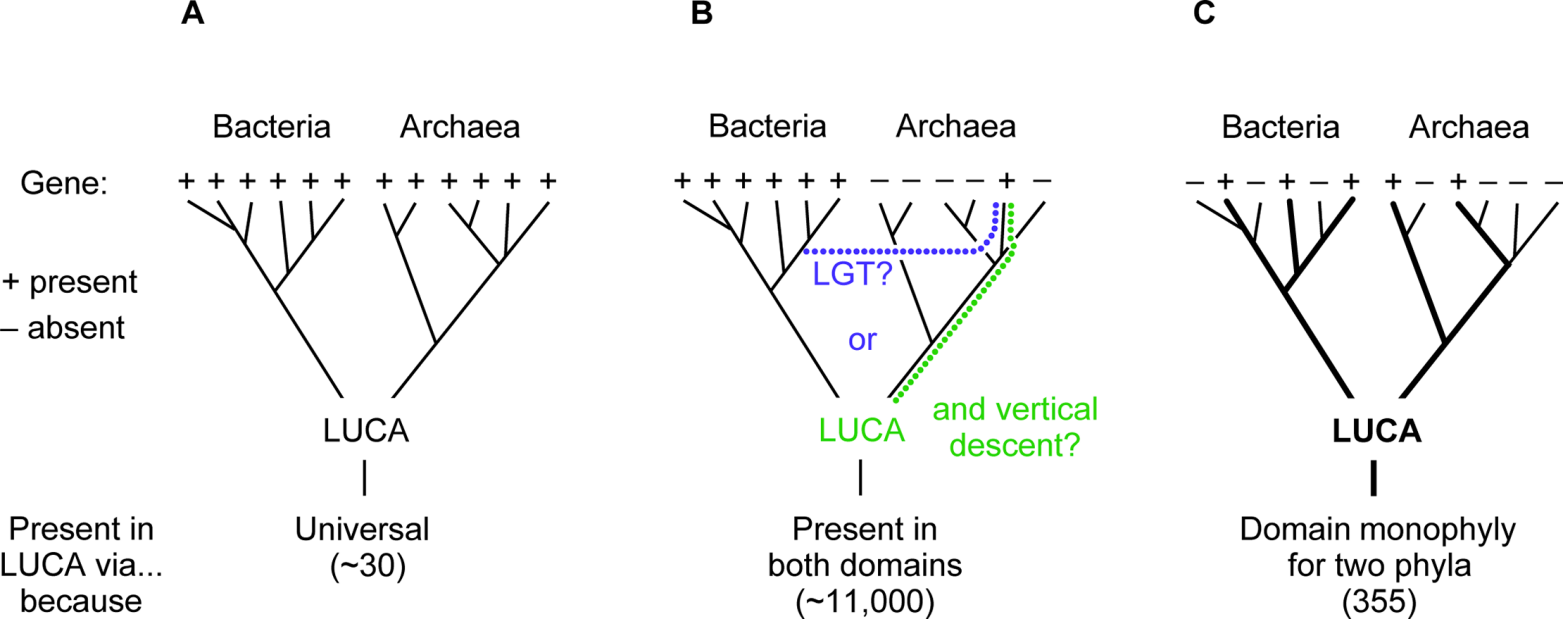
Three ways to infer genes present in LUCA. The gene presence is indicated with a plus sign, absence with a minus sign. a) Genes found universally in both domains, regardless of their tree, trace to LUCA. About 30 fulfil this criterion. b) Another way to trace genes to LUCA is to say that any gene found in both archaea and bacteria was present in LUCA. However, thousands of these genes will have been transferred between bacteria and archaea by LGT so were not necessarily present in LUCA. c) Genes present in only one bacterial or archaeal phylum could easily be the result of LGT and are removed. But presence in two phyla per domain while preserving domain monophyly yields good candidates to have been present in LUCA. Such phylogenies would only result from LGT under very specific and restrictive conditions. They require exactly one transdomain transfer followed by either i) one additional transdomain LGT from the same donor lineage to a different recipient phylum or ii) retention during phylum divergence in the recipient domain, plus—in addition to either criteria i) or ii)—an additional, more subtle but highly restrictive criterion: No further transdomain LGTs occurred during all of evolution. Subsequent transdomain LGT would violate domain monophyly for the gene. Indeed, transdomain LGT is common, and 97% of the trees examined by Weiss and colleagues [78] did not exclude transdomain LGT (remaining 3%, 355 trees, provided in S1 Appendix). LGT, lateral gene transfer; LUCA, last universal common ancestor.

Using the 25% identity threshold, the 6.1 million prokaryotic proteins sampled fall into 286,514 clusters of at least two sequences, and 11,093 of those clusters include sequences found in both archaea and bacteria [78]. Many of those clusters involve oxygen-dependent respiratory chains. Did LUCA have 11,000 genes in its genome and breathe oxygen? That is, was LUCA (and hence archaea) descended from cyanobacteria? Neither prospect seems likely enough to warrant further discussion [85]. Knowing that transdomain LGT is prevalent [54–56] and that thousands of typically bacterial genes are shared with only one archaeal group [58], Weiss and colleagues [78] reasoned that a simple way to exclude some LGTs would be to set the minimal phylogenetic criteria that 1) a gene needs to be present in bacteria and archaea, 2) it needs to be present in at least two phylum-level clades, and 3) the tree needs to preserve domain monophyly (Fig 2). Genes that do not fulfil criterion 1 are not candidates for LUCA anyway. The two-phylum-plus-monophyly criteria 2 and 3 make it less likely but not impossible that such a gene attained that distribution via LGT. How so? Criteria 2 and 3 would require one transdomain transfer followed by intradomain transfers to different phyla, while allowing no subsequent, independent transdomain transfers. The last condition is the restrictive one.

Of the 11,093 clusters that harbored sequences in bacteria and archaea, only 355 (3%) passed the simple LGT filter [78]. Put another way, 97% of the sequences present in bacteria and archaea apparently underwent some transdomain LGT, underscoring the degree to which transdomain LGT has influenced gene history since LUCA and underscoring the need to employ phylogenetic filters in search of genes that trace to LUCA [21,51]. The 97% LGT value is important with regard to the 25% clustering threshold and possible false negatives; 97% of all false negatives founded in low-sequence conservation would still not trace to LUCA because of transdomain LGTs. But transdomain LGT has apparently not erased all signals, as 355 genes passed the LGT test, and those genes tell us things about LUCA that we did not know before.

### The physiology of LUCA

**Fig 3:**
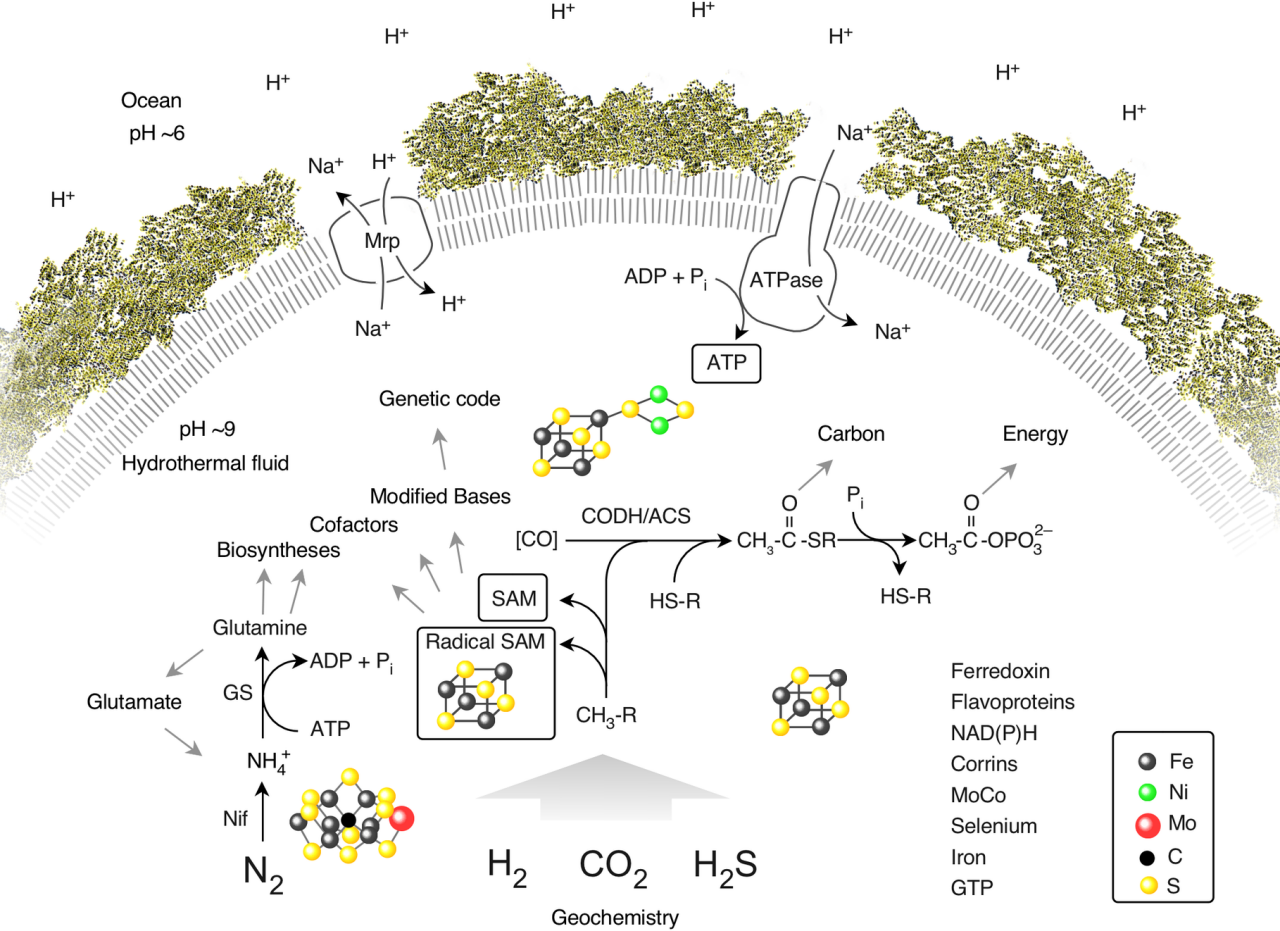
The physiology of LUCA. Summary of the main interactions of LUCA with its environment, reprinted with permission from [78] (supporting trees in S1 Appendix). Components listed at the lower right are present in LUCA. The figure does not make a statement regarding the source of CO in primordial metabolism, symbolized by [CO]. LUCA indisputably possessed genes because it had a genetic code. Transition metal clusters are symbolized. CH_3_-R, methyl groups; CODH/ACS, carbon monoxide dehydrogenase/acetyl–CoA synthase; GS, glutamine synthetase; HS-R, organic thiols; LUCA, last universal common ancestor; Mrp, MrP type Na^+^/H^+^ antiporter; Nif, nitrogenase; SAM, S-adenosyl methionine.

Most earlier depictions of LUCA focused on what it was like [16]; for example, whether it was like RNA [86], like a virus [87], whether it was like prokaryotes in terms of its genetic code [88], or like eukaryotes in terms of its cellular organization [22]. But traditional approaches lacked information about how and from what LUCA lived [16]. Our phylogenetic approach to LUCA [78] uncovered information about what LUCA was doing: its physiology, its ecology, and its environment. The genes for those physiological traits are not necessarily widespread among modern genomes, but the filtering criteria by Weiss and colleagues [78] only require that these genes are ancient. What Weiss and colleagues [78] found is schematically summarized in Fig 3.

LUCA was an anaerobe, as long predicted by microbiologists [89]. Its metabolism was replete with O_2_-sensitive enzymes. These include proteins rich in O_2_-sensitive iron–sulfur (FeS) clusters and enzymes that entail the generation of radicals (unpaired electrons) via S-adenosyl methionine (SAM) in their reaction mechanisms. That fits well with the 50-year-old [90] but still modern view that FeS clusters represent very ancient cofactors in metabolism [91–93]. It also fits with newer insights about the ancient and spontaneous (nonenzymatic) chemistry underlying SAM synthesis [94].

LUCA lived from gasses. For carbon assimilation, LUCA used the simplest and most ancient of the six known pathways of CO_2_ fixation, called the acetyl–CoA (or Wood–Ljungdahl) pathway [95–97], which is increasingly central for our concepts on early evolution because of its chemical simplicity [97,98] and exergonic nature [99–101]. In the acetyl–CoA pathway, CO_2_ is reduced with hydrogen (H_2_) to a methyl group and CO. The methyl group is synthesized by the methyl branch of the pathway, which employs different one-carbon (C1) carriers in bacteria (tetrahydrofolate) and archaea (tetrahydromethanopterin), cofactors that are synthesized by unrelated biosynthetic pathways [96]. Carbon monoxide (CO) is synthesized by carbon monoxide dehydrogenase (CODH), the archaeal and bacterial versions of which are distinct but related [96]. The methyl and carbonyl moieties are condensed to an enzyme-bound acetyl group that is removed from a metal cluster in acetyl–CoA synthase (ACS) as an energy rich thioester. Thioesters harbor chemically reactive bonds [102] that play a crucial role in energy metabolism [101] and in metabolism in general, both modern and ancient [101,103,104]. Although CODH/ACS clearly does trace to LUCA [78,96], this is not true for the methyl synthesis branch, which consists of unrelated enzymes in bacteria and archaea [78,96].

A recent report [105] argued that the presence of CODH in LUCA did not exclude a heterotrophic lifestyle for LUCA. This argument is problematic because no single enzyme defines a trophic lifestyle. Even Rubisco (D-ribulose-1, 5-bisphosphate carboxylase/oxygenase), the classical Calvin cycle enzyme, is not a marker for autotrophy because Rubisco also functions in a simpler heterotrophic pathway of RNA fermentation [106–108] that is common among archaea and bacteria in marine sediment environments [109]. Moreover, all heterotrophs are derived from autotrophs due to the former requiring the latter as a source of chemically defined growth substrates. The reason is that CO_2_ constituted the main carbon source on Earth after the moon-forming impact [1,110], while carbon delivered from space was either too reduced to be fermented (polyaromatic hydrocarbons), too heterogeneous in structure to support microbial growth, or both [108]. Autotrophs with CODH can obtain ATP from CO_2_ reduction with H_2_ [98,101,110]. Autotrophs without CODH cannot. If we base inferences about LUCA's lifestyle on broad criteria rather than single genes [105], LUCA was an autotroph [78,108].

Life is about harnessing energy [44]. Thioesters are chemically reactive—they forge direct links between carbon metabolism and energy metabolism (ATP synthesis) as they give rise to acetyl phosphate, the possible precursor of ATP in evolution as a currency of high-energy bonds [111]. Relics of ATP synthesis via acetyl phosphate were found in LUCA's genes [78], as were subunits of the rotor–stator ATP synthase itself. The ATP synthase might appear to present a paradox because no proteins of the proton-pumping machinery that cells use to generate the ion gradient that drives the ATP synthase traced to LUCA [78]. Yet some theories have it that the first cells arose at alkaline hydrothermal vents [91,96,111], meaning that the inside of the vent is more alkaline than the ocean outside. Such naturally existing pH gradients could have been harnessed by LUCA to synthesize ATP (Fig 3). Ancestral ATPases might have harnessed either proton gradients or sodium gradients generated by proton/sodium (H^+^/Na^+^) dependent antiporters [112], or they might have even been promiscuous for both kinds of ions, similar to the ATPase of modern microbes that live near the thermodynamic limits of life [113].

LUCA's environment was rich in sulfur; thioesters, SAM, proteins rich in FeS and iron–nickel–sulfur (FeNiS) clusters, sulfurtransferases, and thioredoxins were part of its repertoire, as were hydrogenases that could channel electrons from environmental H_2_ to reduced ferredoxin, which is the main currency of reducing power (electrons) in anaerobes [114]. A recent report provided phylogenetic evidence that archaea are ancestrally H_2_-dependent methanogens [62], compatible with an autotrophic, H_2_-dependent lifestyle of LUCA.

LUCA had a reverse gyrase, an enzyme typical of thermophiles, suggesting that LUCA liked it hot. But independent of the reverse gyrase, simple chemical kinetics provide strong evidence in favor of a thermophilic origin for the first cells [115,116]. The reason is that only uncatalysed or inorganically catalysed reactions existed before there were enzymes. Their rates of reaction were lower than the enzymatically catalyzed reactions. Between 0°C and 120°C (the biologically relevant temperature range), organic chemical reaction rates generally increase with temperature [115,116]. Before there were enzymes, high-temperature environments were more conducive to organic chemical reactions than low-temperature environments [115,116]. Taken together, LUCA's requirement for gasses (CO_2_, H_2_, CO, nitrogen [N_2_]), the prevalence of sulfide, its affinity to high temperature and metals, plus an ability to use but not generate ion gradients all point to the same environment: alkaline hydrothermal vents.

In addition to shedding light on physiology, the 355 trees that showed domain monophyly (S1 Appendix) [78] also have another interesting property: they are reciprocally rooted. That is, the bacteria are rooted in an archaeal outgroup and vice versa. Genes present in LUCA contain information about their lineages and about the groups of bacteria and archaea that branched most deeply in each domain. In both cases, the answer was clostridia (bacteria) and methanogens (archaea). Those are strictly anaerobic prokaryotes that use the acetyl–CoA pathway; live from CO_2_, H_2_, and CO; fix N_2;_ and today inhabit hydrothermal environments in the Earth's crust [117–119].

### The onset of genetics

Though the organization of inanimate matter into living cells with genetics can be charted in mathematical terms [120,121], the biochemical details remain elusive. For example, it is controversial whether LUCA had DNA or not [87]. Several DNA-binding proteins trace to LUCA [78], so it would appear that LUCA possessed DNA, but it is unresolved whether LUCA could actually replicate DNA. For LUCA, DNA might just have been a chemically stable repository for RNA-based replication [122].

**Fig 4:**
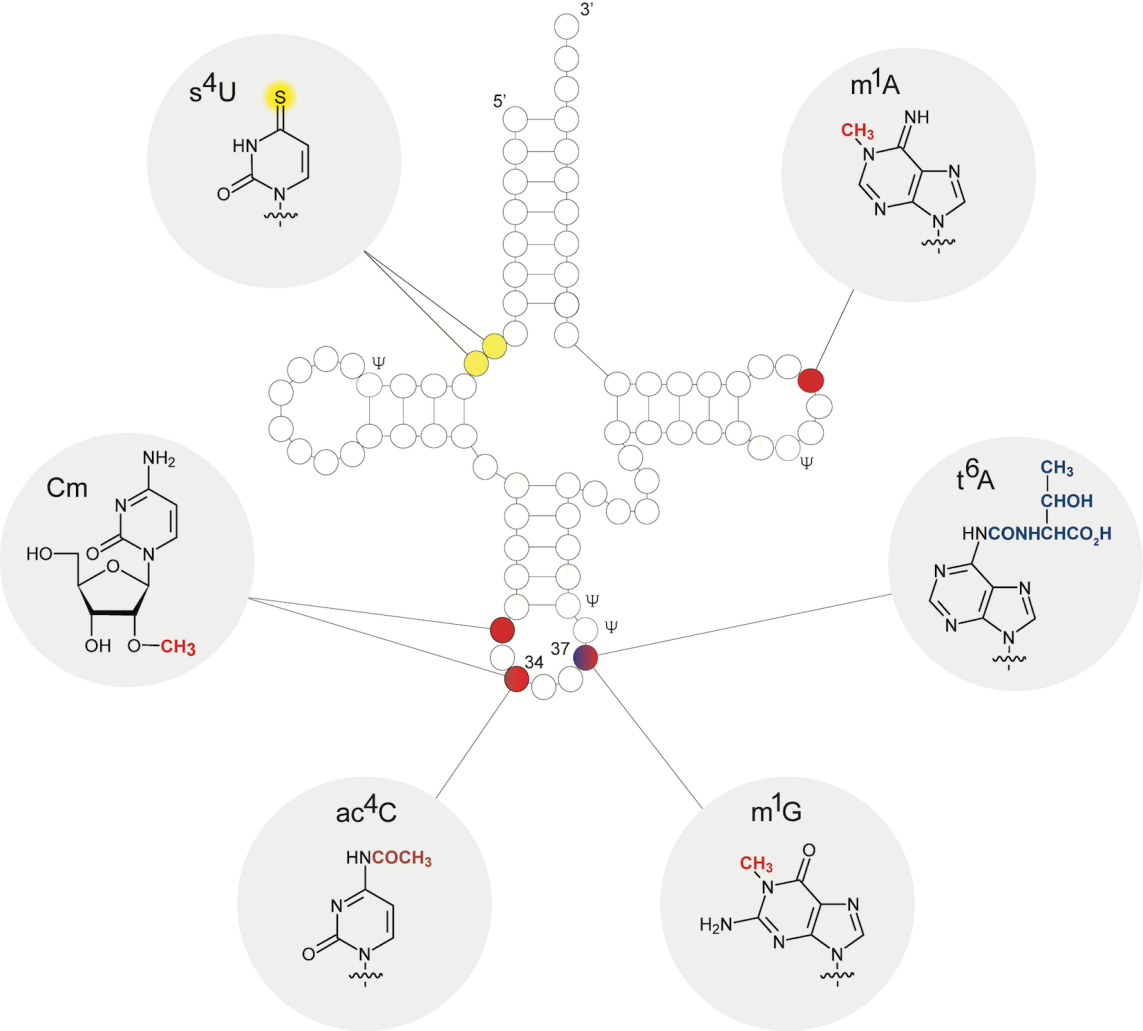
Modified tRNA and nucleoside structures (adapted from [78]). Cloverleaf secondary structure representation of tRNA showing post-transcriptional nucleoside modifications that are conserved among bacteria and archaea in both identity and position. The structures of respective conserved modified nucleosides are highlighted in grey. Methyl and acetyl groups are shown in red and dark red, respectively; sulfur in yellow; and the threonylcarbamoyl group in blue.

A novel and interesting aspect of LUCA's biology concerns modified bases and the genetic code. Transfer RNA requires modified bases for proper interaction with mRNA (codon–anticodon wobble base pairing) and with rRNA in the ribosome during translation. That is, modified bases are part of the universal genetic code (Fig 4), which was present in LUCA. Many RNA-modifying enzymes trace to LUCA, particularly the enzymes that modify tRNA. Several of those enzymes are methyltransferases (many SAM dependent), and they remind us that, before the genetic code arose, the four main RNA bases could hardly have been in great supply in pure form because there were no genes or enzymes, only chemical reactions [123]. Spontaneous synthesis of bases in a real early Earth environment like a hydrothermal vent, an environment that lacks the control of a modern laboratory [124], is not likely to generate the four main bases in pure form. Many side products will accumulate, including chemically modified bases [111]. Chemically modified bases from living cells have been reported since the 1970s by pioneering RNA chemists such as Mathias Sprinzl [125] and Henri Grosjean [126]. There are 28 modified bases, mainly occurring in tRNA, that are shared by bacteria and archaea [127]. The modifications are chemically simple, such as the introduction of methyl groups or sulfur and occasionally of acetyl groups and the like (Fig 4).

Chemical modifications in the tRNA anticodon are essential for codon–anticodon interactions to work [128,129]. Modifications of the rRNA are concentrated around the peptidyl transferase site and are also essential for tRNA ribosome interactions [130]. It is possible that the genetic code itself arose in the same chemically reactive environment where LUCA arose and that modified bases in tRNA carry the chemical imprint of that environment [78]. That would forge a link between the early Earth and genetics as we know it. New laboratory syntheses of RNA molecules in the origin of life context now also include investigations of modified bases [131], as it is becoming increasingly clear that these are crucial components at the very earliest phases of molecular and biological evolution.

### Moving forward

Investigations of LUCA based on phylogenies of all genes pose new opportunities and new challenges. As environmental sequencing and metagenomics progresses, the number of microbial sequences and new lineages is exploding [48,109]. How will that aspect of metagenomics affect investigations of LUCA? If the criteria for gene age are phylogenetic (prokaryote domain monophyly, presence in at least two bacterial and archaeal “phyla”), then the correct taxonomic assignment of each sequence is very important. A problematic aspect of metagenomic data is that some data handling steps can assign incorrect higher taxon labels to genes [39,41,43], which in turn can falsify phylogenetic relationships. Analyses of cultured microbes or complete genome sequences limit the available sample size but deliver reliable taxon labels, at least at the level of archaea versus bacteria. Clearly, there are trade-offs.

At first sight, LUCA's genome appears doomed to shrinkage. As the sample of complete genomes grows, the list of 355 genes that trace to LUCA by domain monophyly criteria [78] will shrink because each new genome offers new opportunities to uncover recent LGT events for the 355 genes. Recalling that only 3% of the 11,093 clusters investigated [78] appeared free of transdomain LGT, it is evident that the inclusion of new genomes will eventually cause the number 355 to asymptotically approach zero, unless some genes never undergo transdomain LGT, which seems unlikely. What to do? Filtering out recent LGT events would help save LUCA's genome from shrinking to zero. For example, the tree for gene X might violate domain monophyly by one LGT event. If the LGT was recent, affecting members of only one recipient genus or family, it would hardly affect inferences about LUCA, adding gene X to LUCA's list. To identify recent LGTs in prokaryote phylogeny, standard criteria like incomplete amelioration [132], anomalously high-sequence identity [133], or presence in the auxiliary genome [134] will be useful, as will new methods that root unrooted trees [135]. Identifying recent LGTs should allow us to trace more genes to LUCA.

There is also the issue of clustering thresholds to consider, as discussed above. Stringent thresholds produce many small clusters and more relaxed thresholds produce a smaller number of very large clusters [136]. One can argue that large clusters (low stringency) allow one to look further back into time, but they also can generate clusters whose origins trace to duplications in LUCA, in which domain monophyly is violated but not because of LGT. Another factor concerns gene fusions. Genes tend to undergo fusion and fission during evolution [137,138]. In clustering procedures, gene fusions tend to slightly reduce the number of clusters because when they occur, they can bring two fused genes into one alignment, and the weaker phylogenetic signal in the fusion is obscured [23]. Methods to detect fusions exist [139,140]. By detecting gene fusions and dissecting them into their component parts, it might be possible to increase the number of trees that trace to LUCA by phylogenetic criteria.

Investigations into early evolution always elicit protest. For example, there were criticisms [141] of the term "progenote," which Woese and Fox [142] introduced to designate a state of organization below that of a free-living cell [143,144], as shown in Fig 3. In addition, multiple LGTs can, in principle, generate false positives by mimicking vertical inheritance from LUCA [78], but very specific conditions have to be fulfilled (Fig 1C). The challenge is to distill a chronicle of microbial evolution that takes all genes and LGT [145] into account and that conveys information about physiology [146], the energy-releasing reactions that power microbial evolution.

### Conclusions

More clues about LUCA's lifestyle are emerging. Investigations of modern biochemical pathways hone in on the same kinds of reactions as the phylogenetic approach [103]. Similarly, laboratory experiments also demonstrate the spontaneous synthesis of end products and intermediates of the acetyl–CoA pathway, the mainstay of LUCA’s physiology; new findings show that formate, methanol, acetyl moieties, and even pyruvate arise spontaneously at high yields and at temperatures conducive to life (30°C–100°C) from CO_2_, native metals, and water [98,147]. Those conditions are virtually impossible to underbid in terms of chemical simplicity [98], yet they bring forth the core of LUCA's carbon and energy metabolism [78,96,97,101,103] overnight. Did the origin of genetics hinge upon hydrothermal chemical conditions that gave rise to the first biochemical pathways that in turn gave rise to the first cells? Genes that trace to LUCA [78], ancient biochemical pathways [103], and aqueous reactions of CO_2_ with iron and water [98,110] all seem to converge on similar sets of simple, exergonic chemical reactions as those that occur spontaneously at hydrothermal vents [148]. From the standpoint of genes, physiology, laboratory chemistry, and geochemistry, it is beginning to look like LUCA was rooted in rocks.

## Supporting information

S1 Appendix ML trees for the 355 protein families that trace to LUCA by phylogenetic criteria. The trees are for the 355 clusters that, after alignment and tree construction, generated ML trees that preserve domain monophyly while also having homologues in ≥2 archaeal and ≥2 bacterial lineages. These 355 proteins trace to LUCA by those phylogenetic criteria [78]. LUCA, last universal common ancestor; ML, maximum likelihood.
(ZIP)
